# The Easiest Way to Drown

**Published:** 1878-05

**Authors:** R. S. Tracy


The Easiest Way to Drown—If death by drowning be inevitable, as in a shipwreck, the easiest way to die would be to suck water into the lungs by a powerful inspiration, as soon as one went beneath the surface. A person who had the courage to do this would probably become almost immediately unconscious, and never rise to the surface. As soon as the fluid filled his lungs, all feelings of chilliness and pain would cease, the indescribable semi-delirium that accompanies anæsthesia would come on, with ringing in the ears and delightful visions of color and light, while he would seem to himself to be gently sinking to rest on the softest of beds and with the most delightful of dreams.—*Dr. R. S. Tracy in Popular Monthly for May.*

